# Diagnostic accuracy study of the VALENF instrument in hospitalization units for adults: a study protocol

**DOI:** 10.1186/s12912-023-01567-4

**Published:** 2023-10-27

**Authors:** Víctor M. González-Chordá, David Luna Aleixos, Irene Llagostera Reverter, Àgueda Cervera-Gash, Francisco Herrero Machancoses, María Teresa Moreno-Casbas, Patricia Flor Arasil, María Jesús Valero Chillerón

**Affiliations:** 1 Nursing Research Group (GIENF-241), Ministerio de Ciencia E Innovación, Universitat Jaume I, Investén-ISCIII, Instituto de Salud Carlos III, Castellón de La Plana, Spain; 2 Nursing Research Group (GIENF-241), Unidad de Hospitalización De Traumatología y Corta Estancia, Hospital Universitario de La Plana, Universitat Jaume I, EnfermeroCastellón de La Plana, Spain; 3 Nursing Research Group (GIENF-241, Universitat Jaume I, Avda Sos Baynat Sn. 12071, Castellón de La Plana, Spain; 4 Predepartamental Unit of Medicine, Universitat Jaume I, Castellón de La Plana, Spain; 5 Investén-ISCIII, Ministerio de Ciencia E Innovación, Instituto de Salud Carlos III, Madrid, Spain; 6 Valencian International University, Valencia, Spain

**Keywords:** Nursing, Nursing assessment, Risk assessment, Hospitalization units, Diagnostic accuracy

## Abstract

Recently, the VALENF instrument, Nursing Assessment by its acronym in Spanish, was developed as a meta-tool composed of only seven items with a more parsimonious approach for nursing assessment in adult hospitalization units. This meta-tool integrates the assessment of functional capacity, the risk of pressure injuries and the risk of falls. The general objective of this project is to validate the VALENF instrument by studying its diagnostic accuracy against the instruments commonly used in nursing to assess functional capacity, the risk of pressure injuries and the risk of falls. An observational, longitudinal, prospective study is presented, with recruitment and random selection based on admissions to six adult hospitalization units of the Hospital Universitario de La Plana. The study population will be made up of patients hospitalized in these units. The inclusion criteria will be patients over 18 years of age with a nursing assessment within the first 24 h of admission and an expected length of stay greater than 48 h and who sign the informed consent form. The exclusion criteria will be transfers from other units or centers. A sample of 521 participants is estimated as necessary. The evaluation test will be the VALENF instrument, and the reference tests will be the Barthel, Braden and Downton indices. Sociodemographic variables related to the care process and results such as functional loss, falls or pressure injuries will be collected. The evolution of functional capacity, the risk of falls and the risk of pressure injuries will be analyzed. The sensitivity, specificity and positive predictive values of the VALENF instrument will be calculated and compared to those of the usual instruments. A survival analysis will be performed for pressure injuries, falls and patients with functional loss. The VALENF instrument is expected to have at least the same diagnostic validity as the original instruments.

**Trial registration** The study will be retrospectively registered (ISRCTN 17699562, 25/07/2023).

## Background

Nurses who work in hospitalization units are responsible for evaluating, planning, executing and reassessing the care that patients require throughout the care process and documenting all of this in the clinical history. However, this group perceives health documentation as an administrative burden due to the increase in the amount of data and the duplication of elements [1]. Moreover, the implementation of electronic health records has prolonged data recording times, increased workloads [2, 3], and reduced direct care times [4] and has made nursing assessments incomplete, inconsistent, and inaccurate [5].

Nursing assessment is defined as a planned, systematic, continuous, and deliberate process of collecting, classifying, and categorizing individualized information to recognize individuals' responses to their actual or potential health problems and needs [6]. These assessments are the basis for making nursing diagnoses and carrying out interventions adjusted to the patient’s needs [7]. Therefore, any error, lack of information or the use of instruments with little validity or reliability can affect the next steps in the nursing process and result in fragmented and incomplete care with repercussions on the quality of care and the development of adverse effects [8].

Some factors that could justify nurses perceiving nursing assessment as an administrative burden [9] include increased patient complexity and a heavy workload [10], the use of different standardized nursing languages [11], and electronic medical records developed according to the paper format and without considering the opinions of nurses [12]. All this leads to a greater amount of data and duplicate items and a diversity of evaluation instruments [1].

Different studies have shown that nursing assessments do not meet adequate standards of quantity or quality of information [13–15], including studies that have analyzed the completion of information on functional capacity, falls or pressure injuries [12, 16–18]. In fact, these instruments are probably the most commonly used by nurses in adult hospitalization units. These instruments are used independently, but they share constructs, dimensions, and items related to mobility, hygiene, eating, or elimination [19, 20], which implies that their items become redundant and are duplicated [1]. The use of redundant assessment instruments generates skepticism and a perception of wasting time, making it difficult for them to be accepted and implemented in nursing [21]. Therefore, nursing assessments can become an automatic and imprecise task without much input from nurses, affecting not only their validity but also the task of detecting patients at risk [22].

Recently, the “VALoración ENFermera” (VALENF) instrument, Nursing Assessment by its acronym in Spanish, was developed as a meta-tool composed of only 7 items with a more parsimonious approach for nursing assessment in adult hospitalization units [23, 24]. This meta-tool integrates the assessment of functional capacity, the risk of pressure injuries and the risk of falls. Thus, the VALENF instrument has a high predictive capacity on the Barthel (R^2^adj = 0.938), Braden (R^2^adj = 0.926) and Downton (R^2^adj = 0.921) indices, with high interobserver reliability (ICC > 0.9) and good construct validity (RMSEA = 0.0726; TLI = 0.968) and internal consistency (Ω = 0.864), although its sensitivity, specificity, and predictive values for detecting patients at risk of functional loss, pressure injuries, or falls remain to be determined. Consequently, the main objective of this project is to study the diagnostic accuracy of the VALENF instrument compared to instruments commonly used in nursing in adult hospitalization units to assess functional capacity, the risk of pressure injuries, and the risk of falls.

## Methods/design

### Design

**Fig. 1:**
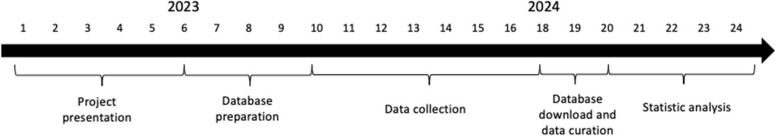
Timeline of the study

An observational, longitudinal, prospective study with recruitment and random selection of hospitalized patients is presented to estimate the diagnostic accuracy of a meta-tool that collapses other instruments for assessing functional capacity and the risk of pressure injuries and falls in a public hospital in the province of Castellón. The study began in January 2023 and will end in December 2024. Figure 1 presents a general timeline of the study.

### Participants and sample

The study population will be made up of patients admitted to the adult hospitalization units of the Hospital Universitario de La Plana. Special service units (intensive care, emergency, operating room and resuscitation), home hospitalization units and maternal-infant and obstetric-gynecological hospitalization units will not be part of the study. Patients over 18 years of age who are assessed in the first 24 h after admission to the hospitalization unit using the Barthel Index, Braden Index, Downton scale and VALENF instrument, are expected to stay longer than 48 h, and who agree to participate in the study and sign the informed consent form will be included in the study. Patients who are transferred from other units of the same hospital and other hospitals will be excluded since their care process is in progress and their assessment on admission does not correspond to the initial assessment after admission.

The sample size was calculated with Epidata v4 and was based on a comparison of proportions for paired data, since the condition of the patient will be unknown at the time of the evaluations, and the reference tests will be used for all subjects. A confidence level of 95%, an accuracy of 80% and a replacement rate of 10% were considered. Thus, the sample size includes 521 participants who will be stratified considering the monthly mean number of discharges from each hospitalization unit (Table 1).

**Table 1:** Sample size estimation

Unit	Discharges in 2019	Mean monthly discharges	Estimated n (8 months)
Trauma 1A – A&E	2023	169	115
Gynecological surgery 1B	1303	109	75
Cardiology-Digestion 1D	1751	146	100
Neurology-Pneumology 2A	1550	129	89
Surgery 2B	1397	116	79
Internal Medicine 2D	1101	92	63
**Total sample**	9125	760	521

### Variables and instruments

The study includes sociodemographic variables (age and sex); variables related to the care process, such as hospitalization unit, type of process (medical, surgical), type of admission (scheduled, urgent), main diagnosis, Charlson Comorbidity Index [25, 26], pressure injury on admission (yes, no), and admission motivated by fall (yes, no); outcome variables that necessitate nursing care, such as falls, pressure injuries, and functional loss [27]; the test to be evaluated (VALENF instrument) and the reference tests (Barthel Index [28], Braden Index [29] and Downton scale [30]).

### Training of nurses

Before starting data collection, the nurses responsible for carrying out measurements will receive training to guarantee homogeneity in data collection. This training program aims to equip them with the necessary skills to use the software used in data collection and standardize patient measurements. To achieve this, several meetings will be held with nurses who voluntarily choose to participate in data collection. During these meetings, the project will be explained in detail, presenting the objectives, participant selection criteria and measurement instruments, as well as the recruitment flow and monitoring of participants during hospitalization. At the end of the training, nurses will be provided with a notebook with the necessary information to serve as a reference at any time during data collection.

### Data collection

Initially, data collection will take place between October 2023 and May 2024 using Research Electronic Data Capture (REDCap) software. Sociodemographic variables and variables related to the care process will be collected only upon admission. Moreover, the test under evaluation (VALENF instrument) and the reference tests (Barthel Index, Braden Index, and Downton scale) will be employed upon admission, every five days, and upon discharge. Finally, the outcome variables sensitive to nursing care will be collected at the time of discharge.

Nurses who work in the hospitalization units that will participate in the study will carry out the recruitment, data collection and follow-up during the hospitalization of the patients included in the study prospectively.

**Fig. 2:**
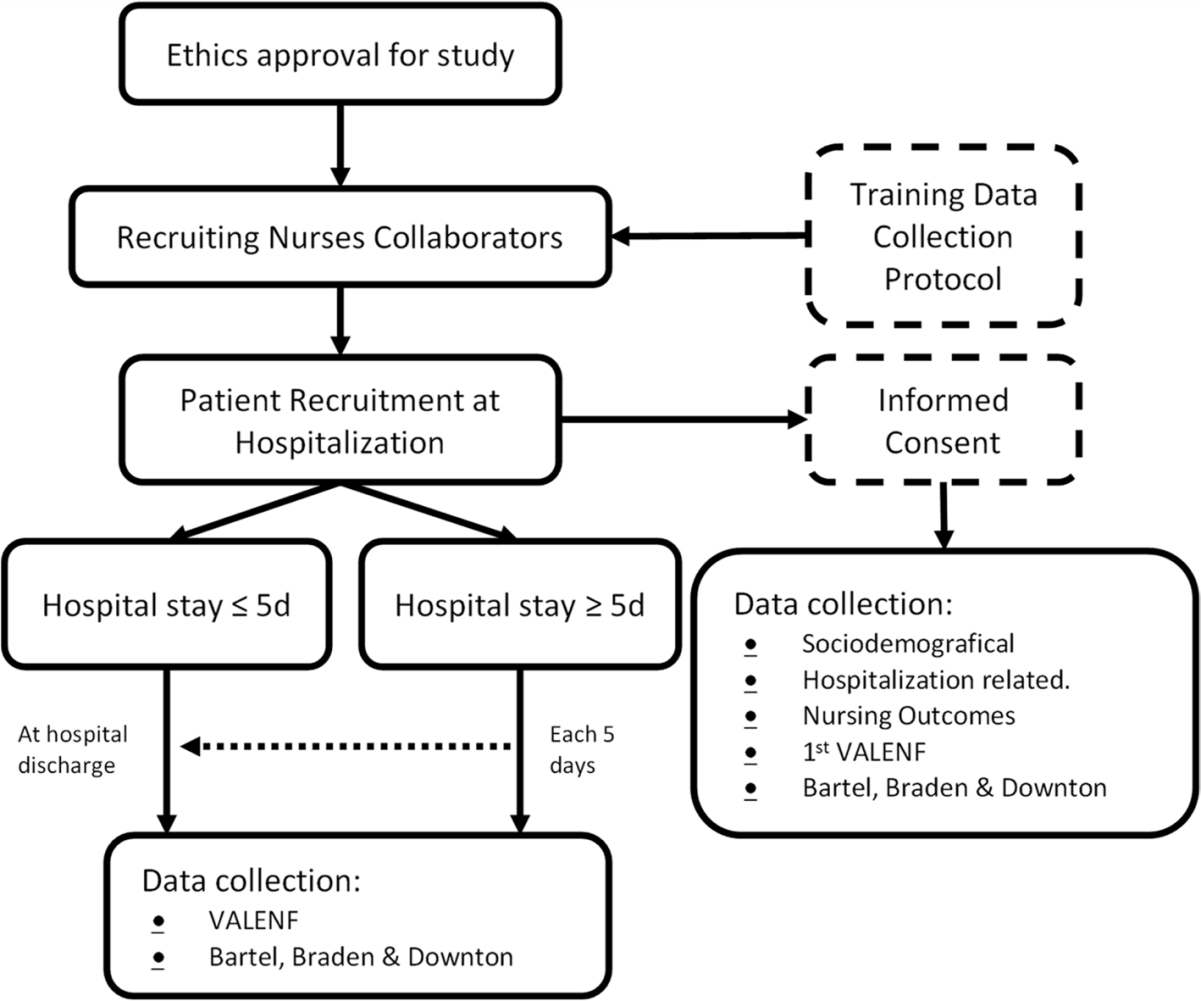
Flow chart with the procedure for recruiting and hospitalization follow-up participants

The recruitment procedure will begin when a patient is admitted to one of the participating hospitalization units, as long as one of the nurses participating in the study is on their work shift during the first 24 h after entry. To randomize the recruitment of the participants, a list of random numbers will be generated for each unit, and a box will be prepared with opaque envelopes with included/not included cards. The nurse who conducts the recruitment will explain the project and request informed consent (IC). The patients will be assessed upon admission, at five days, and upon discharge (Fig. 2).

### Statistical analysis and diagnostic accuracy

First, a descriptive analysis of the sample will be carried out based on the nature of the variables, and whether there are significant differences in the results of the nursing assessments in terms of the hospitalization units, the type of process (medical or surgical), type of admission (urgent or programmed) and the main diagnosis will be evaluated. For this, the normality and homoscedasticity of the sample will be studied with Shapiro‒Wilk and Levene tests, respectively. Depending on the results, Student’s t test or ANOVA will be applied, depending on the number of groups, or the corresponding nonparametric tests. In addition, an analysis of paired measures will be carried out to analyze the evolution of the scores of the Barthel, Braden and Downton indices, what will serve us as a measure of diagnostic accuracy for our instrument, using these measurements as criteria. Categorical variables will be analyzed using the chi-square or Fisher’s exact test, and correlations will be studied with the Pearson or Spearman test, depending on the application conditions.

After this initial analysis, the cumulative incidence and period incidence of pressure injuries, falls and patients with functional loss will be estimated by unit and overall. A survival analysis will be carried out with pressure injuries, falls or functional loss as dependent variables and the other study variables as independent variables.

Next, the sensitivity, specificity, and positive and negative predictive values of the VALENF instrument will be analyzed to detect patients at risk of pressure injuries, falls, and functional loss using the Barthel, Braden, and Downton indices as reference tests. At this point, it should be considered that the protocolized prevention measures will be applied for those patients who are assessed to be at risk of pressure injury or fall to safeguard the ethical and deontological commitment of nonmaleficence. For this reason, this analysis will be carried out only with the positive cases first and, second, including the true positives with those other patients to whom the corresponding prevention measures were applied. We understand that this situation may bias the diagnostic accuracy results of the VALENF instrument, but we believe that it is a viable solution and that it can also provide information on the effectiveness of the measures used.

Finally, the VALENF instrument cutoff points will be established to detect the risk of pressure injuries, falls, and functional loss through simple linear regressions with the original instruments and cluster analysis to categorize patients. Moreover, and analysis of the area under the ROC curve will be carried out, considering acceptable values 0.7 to 0.8, excellent if 0.8 to 0.9, and outstanding if more than 0.9, and the Youden index will be used to calculate the optimal cut-off point [31]. A value of *p* < 0.05 will be considered in the comparisons of the hypotheses, and the statistical analysis will be carried out with SPSS v-25 and Jamovi v-2.3.2 software.

### Ethical considerations

The project was positively evaluated by the Ethics and Research Committee of the participating hospital in July 2023. This project complies with Regulation (EU) 2016/679 of the European Parliament and of the Council of 27 April, 2016, regarding the protection of natural persons and Organic Law 3/2018 of 5 December, on Protection of Personal Data and Guarantee of Digital Rights. The participants will receive adequate and timely information about the objective and methodology of the study, as well as about their rights to access the information and to abandon the study. This information will be provided by the nurses who participate in the study. After being informed, they will provide informed consent with their signature. The database will not include personal data that allow the identification of patients.

## Discussion

The limitations of this study mainly involve sample selection bias and measurement bias due to misclassification of the subjects due to the subjectivity of the measurement and underreporting of results sensitive to nursing practice. Another important aspect to highlight is the risk of patient loss during hospitalization follow-up and discharge.

To try to control these limitations, a team of nurses who participate voluntarily in the study and who will receive specific training on the objective and methodology of the study will be selected. Adequate training on REDCap will be carried out, and standardized measurements will be used, verifying compliance with the requirements through a CDRe pilot. In addition, during data collection, the researchers are expected to visit the units periodically to resolve doubts and problems related to the recruitment, data collection, and hospitalization follow-up of participants, and three researchers will audit the REDCap database to verify its accuracy and correct completion and reduce the risk of loss.

Finally, patients at risk (falls or pressure injuries) need preventive measures to avoid these undesirable results. These measures will be applied when necessary and considered in the CDR and data analysis.

## Data Availability

The datasets generated and/or analyzed during the current study are not publicly available due to this being a protocol study but will be made available from the corresponding author upon reasonable request.
